# Loss of αA or αB-Crystallin Accelerates Photoreceptor Cell Death in a Mouse Model of P23H Autosomal Dominant Retinitis Pigmentosa

**DOI:** 10.3390/ijms23010070

**Published:** 2021-12-22

**Authors:** Tiantian Wang, Jingyu Yao, Lin Jia, Patrice E. Fort, David N. Zacks

**Affiliations:** 1 Department of Ophthalmology and Visual Sciences, University of Michigan, Ann Arbor, MI 48105, USA; tiantiaw680@gmail.com (T.W.); yjingyu@med.umich.edu (J.Y.); ljia@med.umich.edu (L.J.); patricef@med.umich.edu (P.E.F.); 2 Department of Ophthalmology, Xiangya Hospital, Xiangya School of Medicine, Central South University, Changsha 410008, China

**Keywords:** α-crystallin, P23H, autophagy, cell death, microglia

## Abstract

Inherited retinal degenerations (IRD) are a leading cause of visual impairment and can result from mutations in any one of a multitude of genes. Mutations in the light-sensing protein rhodopsin (RHO) is a leading cause of IRD with the most common of those being a missense mutation that results in substitution of proline-23 with histidine. This variant, also known as P23H-RHO, results in rhodopsin misfolding, initiation of endoplasmic reticulum stress, the unfolded protein response, and activation of cell death pathways. In this study, we investigate the effect of α-crystallins on photoreceptor survival in a mouse model of IRD secondary to P23H-RHO. We find that knockout of either αA- or αB-crystallin results in increased intraretinal inflammation, activation of apoptosis and necroptosis, and photoreceptor death. Our data suggest an important role for the ⍺-crystallins in regulating photoreceptor survival in the P23H-RHO mouse model of IRD.

## 1. Introduction

Inherited retinal degeneration (IRD) is a significant cause of progressive, irreversible blindness throughout the world. Mutations in over 300 genes have been identified as causing IRD [1,2], and transmission in autosomal dominant, autosomal recessive, and x-linked patterns have been described [3,4,5]. Autosomal dominant retinitis pigmentosa (adRP) derives from inherited mutations in a variety of retinal genes, and, similar to other IRDs, results in progressive photoreceptor cell death and vision loss [6,7]. Mutations in the light-sensing protein, rhodopsin (RHO; encoded for by the RHO gene) is a leading cause of adRP, with the most common of those being a missense mutation that results in substitution of proline-23 with histidine. This rhodopsin variant, also known as P23H-RHO, has been extensively studied, as it is the most frequent cause of adRP in the United States [8,9,10,11].

The P23H variant results in rhodopsin misfolding, initiation of endoplasmic reticulum stress (ERS) and the unfolded protein response (UPR), and activation of cell death pathways [12,13,14]. Multiple death pathways have been implicated to participate in the loss of P23H-RHO containing photoreceptors, including autophagy, apoptosis, and necroptosis [15,16,17,18,19,20]. We have previously shown that the presence of P23H-RHO results in a relative insufficiency of proteasome activity and a concomitant increase in autophagy flux and a resultant increase in autophagic cell death [21]. We found that interventions that reduce autophagy flux or increase proteasome activity can reduce the autophagic cell death and increase photoreceptor survival [22]. These findings suggest that molecular chaperones that help correct the folding of rhodopsin and facilitate the degradation of the P23H-RHO by the proteasome may restore photoreceptor proteostasis and reduce autophagic cell death.

The alpha (α) family of crystallins are soluble small molecular proteins, divided into αA-crystallin and αB-crystallin. Although α-crystallins were initially thought to only exist as a structural component of the lens, it has subsequently been found that they are members of the small heat shock protein (sHSP) family, and act as molecular chaperones in cells that can reduce the aggregation of misfolded proteins [23,24,25,26]. Their function is highly controlled by post-transcriptional modifications. For example, we recently showed that a specific phosphorylation site of αA-crystallin which reduced ERS induced by metabolic stress was shown to be dramatically reduced in retinal tissues from diabetic retinopathy donors [27]. Others have reported the phosphorylation of αB-crystallin enhances its chaperone function of vascular endothelial growth factor to modulate angiogenesis [28]. Furthermore, α-crystallins can exhibit neuroprotective properties in preventing or rescuing retinal damage in various mouse models of retinal injury [29,30,31,32,33,34,35].

In this study, we investigate the effect of ⍺A-crystallin or ⍺B-crystallin knockout on photoreceptor survival in a mouse model of adRP secondary to P23H-RHO. In particular, we test the hypothesis that loss of either of these crystallins will result in increased photoreceptor cell death relative to P23H-RHO alone. We crossed mice with systemic knockout of either ⍺A-crystallin knockout (AKO) or ⍺B-crystallin knockout (BKO) with P23H mice to generate AKO/P23H and BKO/P23H mice, respectively. We observed that depletion of ⍺-Crystallin (⍺A or ⍺B) in the P23H worsened photoreceptor degeneration. It increased apoptotic and necroptotic photoreceptor cell death, and further elevated inflammation and microglia activation in the P23H retina. However, contrary to our expectation, ⍺-crystallin (⍺A or ⍺B) deficiency did not alter P23H-RHO protein folding or autophagy activation in the photoreceptors of P23H-RHO mice. These results suggest that ⍺-crystallin exerts its protective roles on photoreceptor survival in the P23H-RHO by regulating the activation of inflammation and cell death pathways. These data suggest that α-crystallin pathways are important for photoreceptor survival in adRP secondary to P23H-RHO expression.

## 2. Results

### 2.1. Increased Photoreceptor Cell Death in AKO/P23H and BKO/P23H Mouse Retina

**Figure 1:**
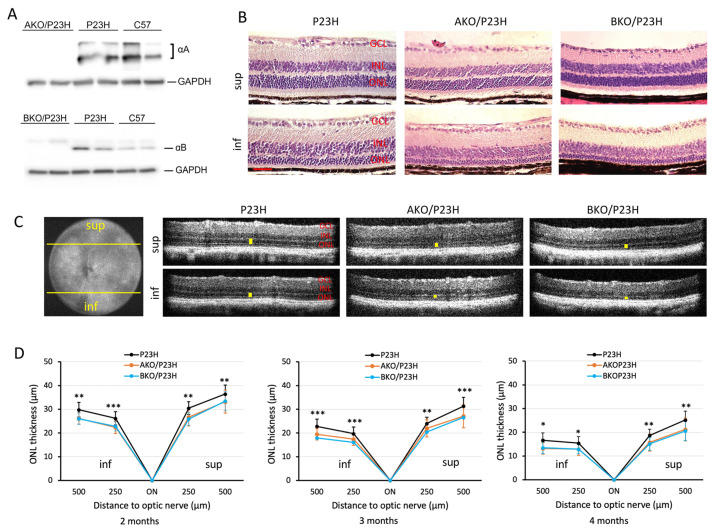
Increased photoreceptor loss in AKO/P23H and BKO/P23H mouse retina compared with age matched P23H controls. (**A**) Representative images of immunoblots probing for αA-crystallin (αA) or αB-crystallin (αB) from whole retinal lysates of AKO/P23H or BKO/P23H, P23H, and C57 confirm the loss of these proteins in the respective strains. (**B**) Representative H&E staining images of superior (sup) and inferior (inf) retina at the age of 4 months in AKO/P23H, BKO/P23H, and control P23H mice show increase loss of photoreceptors in the former compared to the control. (**C**) Representative optical coherence tomography (OCT) images of superior and inferior retina of 4-month-old AKO/P23H, BKO/P23H, and P23H mice correspond to the histology findings in panel B. (**D**) Quantification of the thickness of the outer nuclear layer (indicated by yellow bars in the OCT images) of superior and inferior retina measured at both 250 and 500 µm from the optic nerve head by OCT in AKO/P23H, BKO/P23H, and P23H mice at the age of 2, 3, and 4 months. Scale bar: 50 µm. (n ≥ 16), * *p* < 0.05; ** *p* < 0.01; *** *p* < 0.001; One-way ANOVA. GCL, ganglion cell layer; INL, inner nuclear layer; ONL, outer nuclear layer; RPE, retinal pigment epithelium.

We first confirmed the genetic depletion of the ⍺A- and ⍺B-crystallin protein from the AKO/P23H and BKO/P23H strains (Figure 1A). The P23H mouse retina has differential rates of retinal degeneration in the superior versus inferior retina, with the inferior retina degenerating more rapidly [36]. By HE staining, we detected marked thinning of the outer nuclear layer (ONL), where the photoreceptor nuclei reside, especially in the inferior half of the retina, in both AKO/P23H and BKO/P23H as compared to age matched P23H control mice (Figure 1B). In vivo measurement of the thickness of the ONL by optical coherence tomography (OCT) was performed. There was increased thinning of the ONL, especially in the inferior half of the retina, in both AKO/P23H and BKO/P23H as compared to age-matched P23H control mice (Figure 1C). This increased thinning of the ONL was observed at all age points tested, up to 4 months (Figure 1D). Measurements beyond 4 months of age were limited by cataract formation. There was no difference in ONL thickness, as measured by OCT, between AKO/P23H mice and BKO/P23H mice (Figure 1D). These findings demonstrate that both αA- and αB-crystallin plays protective roles in photoreceptor survival in the P23H mouse retina.

### 2.2. Reduced Rod-Photoreceptor Function in AKO/P23H and BKO/P23H Mouse Retina

**Figure 2:**
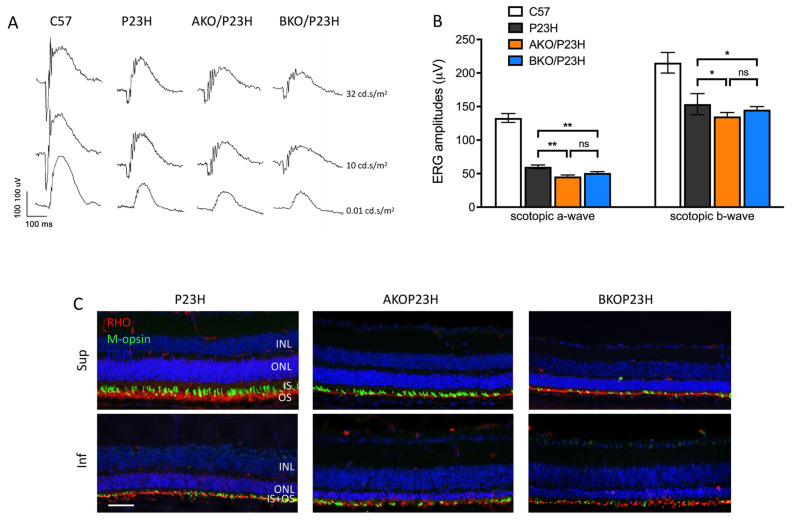
Decreased retinal function in AKO/P23H and BKO/P23H mice. (**A**) Representative scotopic ERG traces at indicated intensity in AKO/P23H, BKO/P23H, P23H and wild-type C57 mice at 4 months of age. (**B**) Quantification of amplitudes of scotopic a-wave and b-wave at the intensity of 10 cd s/m^2^ at 4 months of age confirms the reduction in retinal function corresponding to the increase in photoreceptor cell death. (**C**) Representative immunofluorescent staining images of superior and inferior retina of four-month-old AKO/P23H, BKO/P23H, and P23H mice, stained for rhodopsin (RHO in red), m-opsin(green), and 4′,6-diamidino-2-phenylindole (DAPI in blue) confirm the reduction in photoreceptive elements corresponding to the increase in photoreceptor cell death. Scale bar: 50 µm. (n = 12), * *p* < 0.05; ** *p* < 0.01; ns, not significant; one-way ANOVA. INL, inner nuclear layer; ONL, outer nuclear layer; IS, inner segment; OS, outer segment.

The visual function of AKO/P23H or BKO/P23H mice versus P23H mice was analyzed monthly out to 4 months of age by scotopic electroretinogram (ERG), representing function of rod photoreceptors. Consistent with the decreased ONL thickness and reduced number of photoreceptors, the amplitudes of both the a-wave and the b-wave in AKO/P23H and BKO/P23H mice were significantly diminished compared with P23H mice (Figure 2A,B). There was no significant difference in ERG response between AKO/P23H and BKO/P23H mice (Figure 2B). Immunohistochemical analysis revealed decreased levels of rhodopsin and cone opsin in AKO/P23H and BKO/P23H mice (Figure 2C). These data suggest that the loss of photoreceptor cells in αA- or αB-crystallin depleted P23H mice is accompanied by further reduction in visual function. Taken together, our findings support the view that α-crystallins contribute to the survival and function of photoreceptors in the P23H.

### 2.3. Increased Activation of Cell Death Pathways in AKO/P23H and BKO/P23H Mouse Retina

**Figure 3:**
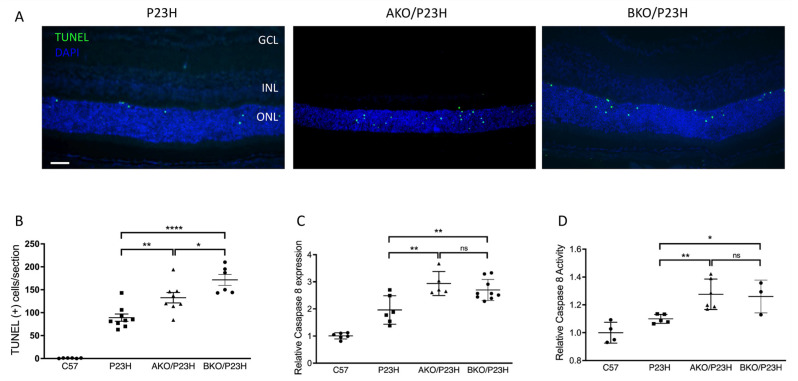
Increase in activation of apoptosis in the photoreceptors of AKO/P23H and BKO/P23H mice. (**A**) Representative TUNEL (green) staining images for AKO/P23H, BKO/P23H, and P23H mice at P14. Nuclei were counterstained with DAPI (blue). (**B**) Quantification of TUNEL-positive cells in the ONL of AKO/P23H, BKO/P23H, and P23H mice. Three non-continuous sections crossing the optic nerve head were used for the quantification of each eye. (**C**) Quantification of transcript levels of caspase 8 in the retinas of AKO/P23H, BKO/P23H, and P23H mice at two months of age, normalized to C57 mice. (**D**) Quantification for caspase 8 activity in the retina of AKO/P23H, BKO/P23H, and P23H mice at two months of age, normalized to C57 mice. (n ≥ 6), * *p* < 0.05; ** *p* < 0.01; **** *p* < 0.0001; ns, not significant. One-way ANOVA. Scale bar: 50 µm. GCL: ganglion cell layer, INL: inner nuclear layer, ONL: outer nuclear layer.

Apoptosis and necroptosis have been found to be involved in photoreceptor cell death in P23H retina [17,18]. We assessed whether the deletion of ⍺A-or ⍺B-crystallin would affect the level of activation of these two pathways. Terminal deoxynucleotidyl transferase dUTP nick end labeling (TUNEL) staining of photoreceptors in the P23H mouse retina peaks at approximately two weeks of age (P14) [22]. We assessed retinal sections from mice at P14 and observed that deletion of αA-crystallin in P23H mice caused approximately a 1.6-fold increase in the number of TUNEL-positive cells in the ONL as compared to controls, while αB-crystallin deletion resulted in a 2-fold increase (Figure 3A,B). In the caspase-dependent apoptotic pathway, initiator caspase-8 activation is a critical upstream event that results in activation of the executioner caspase-3 [37]. We found significant increases in the transcript level of caspase 8 (Figure 3C), as well as a significant increase in caspase 8 activity (Figure 3D) in AKO/P23H and BKO/P23H mice by two months of age compared with age-matched P23H controls. At one month of age, the difference was trending higher, but did not reach statistical significance. The findings confirm the participation of α-crystallin in apoptosis regulation in P23H mice.

**Figure 4:**
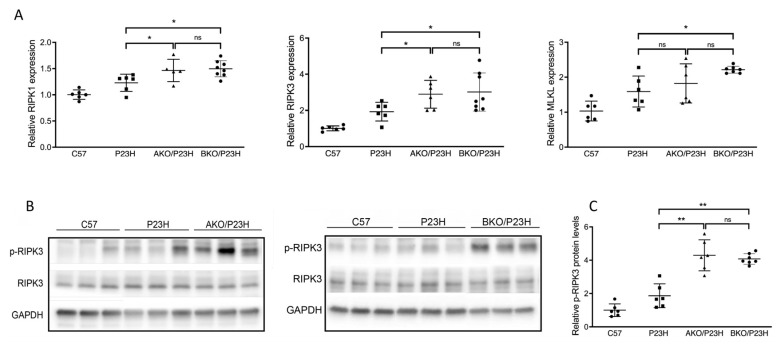
Increase in activation of necroptosis pathway in the photoreceptors of AKO/P23H and BKO/P23H mice. (**A**) Quantification of transcript levels of RIPK3, RIPK1, MLKL in retinas of AKO/P23H, BKO/P23H and P23H mice at two months of age, normalized to levels in the retinas of wild-type C57 mice. (**B**) Representative western blots from retinas of AKO/P23H, BKO/P23H, P23H and C57 mice probed for *p*-RIPK3, RIPK3 and GAPDH. (**C**) Quantification of western-blot bands of *p*-RIPK3 normalized to GAPDH (n = 6), * *p* < 0.05; ** *p* < 0.01; ns, not significant. One-way ANOVA.

Among critical markers of necroptotic cell death, we observed increased transcript levels of receptor interacting serine/threonine kinases 1 and 3 (RIPK1 and RIPK3) in AKO/P23H compared to controls (Figure 4A). In the BKO/P23H mouse retina, we observed increased transcript levels of RIPK1, RIPK3, and mixed lineage kinase domain-like (MLKL) effector of necroptosis (Figure 4A). Increased phosphorylation of RIPK3 (p-RIPK3) protein levels were also observed in both AKO/P23H and BKO/P23H mice compared to P23H controls, consistent with activation of necroptotic cell death (Figure 4B,C). These findings are consistent with the finding that depletion of ⍺-crystallin (⍺A or ⍺B) in the P23H mouse retina results in increased photoreceptor cell death, and are consistent with the hypothesis that α-crystallins modulate the activation of multiple cell death pathways.

### 2.4. Autophagy Activation Unchanged in the AKO/P23H and BKO/P23H Mouse Retina 

**Figure 5:**
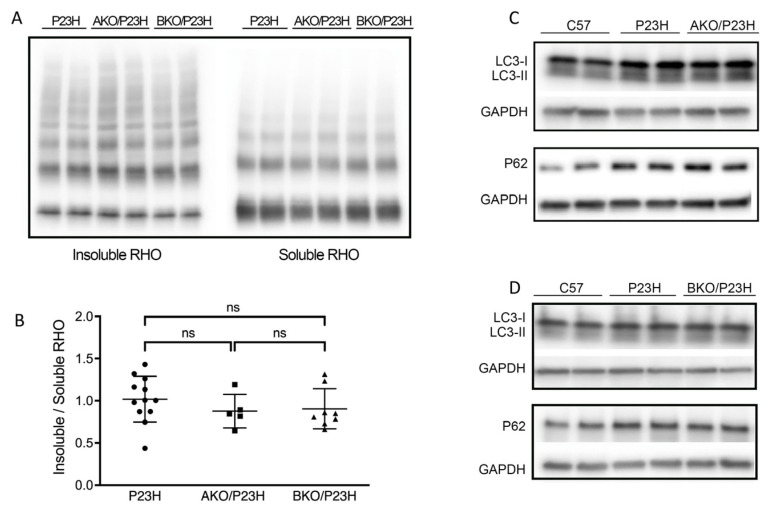
There is no change in the level of soluble P23H-rhodopsin or autophagic activity in the AKO/P23H and BKO/P23H mouse retina. (**A**) Representative western blots probed for rhodopsin (RHO) in the soluble and insoluble fraction of total retinal protein from AKO/P23H, BKO/P23H and P23H mice at the age of two months. (**B**) Quantification of western-blot bands of the ratio of insoluble RHO to soluble RHO. (**C**) Representative western blots probed for LC3 and SQSTM1/p62 and GAPDH in the retina of AKO/P23H, (**D**) BKO/P23H, P23H and C57 mice. For better detection of autophagy flux, mice received an intraperitoneal injection of leupeptin (40 mg/kg body weight) 4 h before sampling. (n ≥ 5), ns, not significant. One-way ANOVA.

Our previous work has shown that the rhodopsin misfolding in P23H-RHO results in increased activation of autophagy and increased autophagic cell death [21]. We also found that chemical chaperone treatment improves P23H-RHO folding, decreases ERS and autophagic flux, and increases proteasome activity [22]. Since α-crystallin is best known for its chaperone activity to correct the misfolded proteins and improve their trafficking to the proteasome [38,39,40], we postulated that they participate in the correction of misfolded rhodopsin and attenuate the autophagic flux in P23H mice. This postulate would predict that deletion of the α-crystallins in the P23H-RHO retina would result in increased rhodopsin aggregate formation and increased autophagy activation [41]. Contrary to our prediction, however, there was no significant reduction in the ratio of insoluble/soluble rhodopsin, a marker of rhodopsin aggregate formation [41], in AKO/P23H and BKO/P23H mice compared to P23H controls at two months of age (Figure 5A,B). We then assessed the level of the classical markers of autophagic activation, including SQSTEM1/p62 and Microtubule-associated protein 1A/1B-light chain 3 (LC3). Of note, p62 has been reported to be a mediator between autophagic activation and proteasome degradation [42,43]. The conversion of LC3-I to LC3-II indicates the increase of autophagosome formation, representing autophagy activation [44]. Again, contrary to our prediction, there was no detectable increase in protein expression of p62 or conversion of LC3I to LC3-II in AKO/P23H and BKO/P23H mouse retinas, as compared to P23H controls (Figure 5C,D).

### 2.5. Cytokine and Microglia Activation in the P23H, AKO/P23H, and BKO/P23H Retina

**Figure 6:**
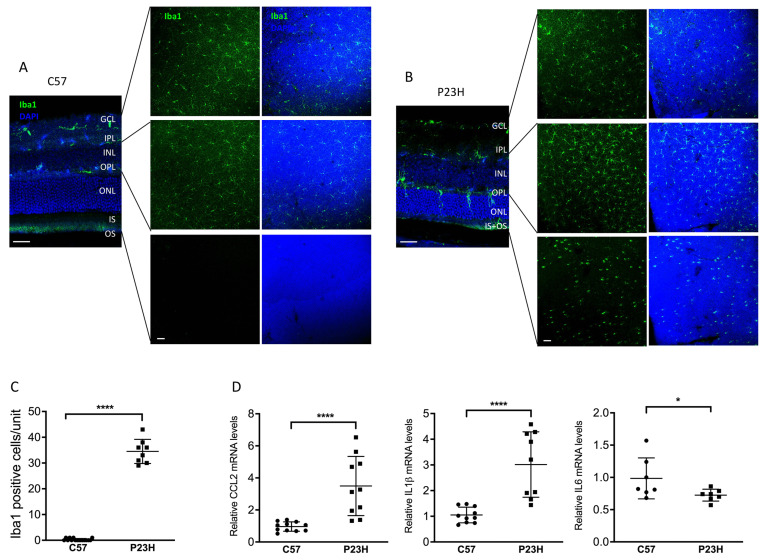
Activated inflammatory cytokines and microglia in the P23H retina. (**A**) Representative confocal images of 30 μm retinal sections and retinal whole mount of two-month-old C57 and (**B**) P23H mouse stained with Iba1 showing Iba-1 positive cells in different layers of the retina as indicated. Nuclei were counterstained with DAPI. (**C**) Quantification of Iba-1-positive cells in the ONL and subretinal space of the inferior area of the retinal whole mount from P23H and C57 mice. Counting unit is confocal image at 40× magnification (**D**) Transcript levels of inflammatory cytokines-CCL2, IL1b and IL6 in the retinas of P23H at two months of age, normalized to C57 mice (n ≥ 7). * *p* < 0.05; **** *p* < 0.0001; one-way ANOVA. GCL, ganglion cell layer, IPL, inner plexiform layer, INL, inner nuclear layer, ONL, outer nuclear layer; IS/OS, inner and outer segment. Scale bar: 50 µm.

Previous studies have shown that inflammation is a key factor in retinal disease progression and the expression of inflammatory cytokines has been shown to be elevated in a variety of retinal diseases [45,46,47]. These cytokines play key roles in the activation and recruitment of microglia, macrophages, and other immune cells to the areas of damaged retina. It has been reported that there exists a persistent inflammatory state in a rat model of P23H [47]. However, inflammatory cytokines in P23H mouse models have not been reported. Consistent with those previous findings [47], we detected activation and migration of the Iba1 labeled immune cells from the inner retina to the ONL as well as subretinal space of the P23H mice (Figure 6A–C). Notably, these Iba1-positive cells showed shortened ramifications and an amoeboid morphology, suggestive of microglial activation. We also assessed gene expression of inflammatory cytokines and found elevated levels of C-C motif chemokine ligand 2 (CCL2), C-C motif chemokine ligand 3 (CCL3), and interleukin-1β (IL-1β) (Figure 6D), consistent with an activated inflammatory state in the P23H retina. Interestingly, this was accompanied by a decreased level of IL-6 in the retinas of P23H mice compared with C57 controls. The reason for this decrease in IL-6 is unclear, but may represent a reduction in its protective function [48].

**Figure 7:**
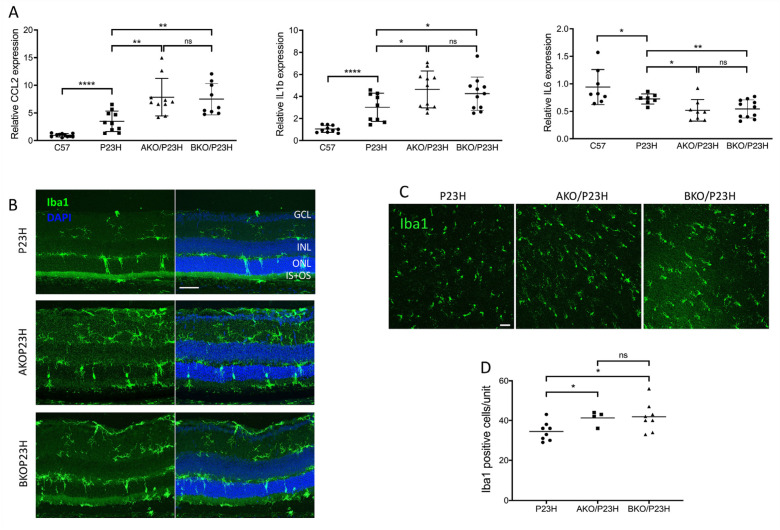
Further increased inflammation in the AKO/P23H and BKO/P23H mouse retina. (**A**) Transcript levels of inflammatory cytokines CCL2, IL1b, and IL6, in retinas of AKO/P23H, BKO/P23H, and P23H mice at two months of age, normalized to levels in the wild-type C57 mice (n ≥ 7). (**B**) Representative immunostaining images of retinal sections from inferior retinas of AKO/P23H, BKO/P23H, and P23H mice at two months of age stained with Iba-1 and DAPI. (**C**) Representative images for ONL from retinal whole mount of AKOP23H, BKOP23H and P23H mice stained with Iba1 at two months of age. (**D**) Quantification of Iba1-positive cells in the ONL and subretinal space of the inferior retina of AKO/P23H, BKO/P23H and P23H mice. (n ≥ 4), * *p* < 0.05; ** *p* < 0.01; **** *p* < 0.0001; ns, not significant. One-way ANOVA. GCL, ganglion cell layer, IPL, inner plexiform layer, INL, inner nuclear layer, ONL, outer nuclear layer; IS/OS, inner and outer segment. Scale bar: 50 µm.

The α-crystallin family has been reported to participate in the modulation of inflammation within the retina [49,50]. In both AKO/P23H and BKO/P23H mice, we detected a further increase in the transcript levels of CCL2 and IL-1β and a further decrease in expression of IL-6, as compared to P23H controls (Figure 7A). To assess the effects of α-crystallin depletion on microglial activation and migration in P23H mice, we performed Iba-1 labelling on both retinal sections (Figure 7B) and retinal whole mounts (Figure 7C) of the AKO/P23H, BKO/P23H, and P23H control mice. Consistent with the increased inflammatory cytokine levels detected, quantification of the total number of Iba1-positive cells in the photoreceptor layer and the subretinal area at two months of age showed significantly increased cell numbers in both AKO/P23H and BKO/P23H mice compared to P23H mice (Figure 7D). 

## 3. Discussion

Though typically studied for their structural role in the ocular lens and maintenance of its transparency, it has become increasingly recognized that crystallin proteins play important other functions, including in the retina. In particular, α-crystallins belong to the small heat shock proteins and have gained attention for their neuroprotective properties [23,50,51] Over the last decade, several groups have demonstrated that lack of one or both α-crystallin proteins was associated with increased neurodegeneration in diabetic retinopathy, endophthalmitis, uveitis, or retinal tear [52,53]. More recently, intervention studies further supported the neuroprotective potential of α-crystallins by demonstrating that either overexpression or exogenous administration was highly protective [54,55]. However, to this day, the potential role of αA- and αB-crystallin in photoreceptor degeneration in retinitis pigmentosa remained unknown. In this study we examine the role α-crystallins play in protecting photoreceptor cells in a model of autosomal dominant retinitis pigmentosa caused by a mutation in the rhodopsin gene that results in the misfolding of the rhodopsin protein. This rhodopsin misfolding results in increased ER stress and activation of death pathways, including apoptosis, necroptosis, and hyperautophagy. We found that deletion of either ⍺A- or ⍺B-crystallin results in increased activation of apoptosis and necroptosis and more rapid photoreceptor cell death.

The mechanism by which rhodopsin misfolding results in photoreceptor cell death is multifactorial, and still not completely understood. It is established that apoptosis and necroptosis are both activated by the P23H-RHO variant, and that these pathways contribute to photoreceptor degeneration. In both AKO/P23H and BKO/P23H mouse retinas, we observed increased TUNEL staining and activation of caspase-mediated apoptosis, as well as increased activation of necroptosis. It is not clear the degree to which each of these contribute to the death of the photoreceptors, i.e., is it primarily apoptotic or necroptotic cell death? The increased activation of apoptosis relative to necroptosis, however, suggests that the former is likely the prevailing death pathway, which is similar to what is found in other models of photoreceptor death such as retinal detachment [56]. A key future area of investigation is whether and how the ⍺A- and ⍺B-crystallin regulate death pathway activity.

One mechanism by which the ⍺-crystallins could exert a protective effect is through their ability to act as a small molecule chaperone. We have previously shown that a small molecule chaperone can improve rhodopsin solubility and increase shuttling of the rhodopsin to the proteasome degradation pathway [22]. The data presented here do not show an increase in the insoluble fraction of rhodopsin upon deletion of either ⍺A- and ⍺B-crystallin. However, it cannot be fully excluded that any increase in the insoluble content remained undetected because of the level of sensitivity of our assay, or the exact timing of our analysis. Indeed, the lack of effect detected in the KO mice could be in part due to an effect on the timeline of the disease. Another potential reason could be that there was compensation in chaperone function by other crystallins or by other proteins, but that this compensatory chaperone activity was independent of the ⍺-crystallin effect on death pathway activation. Future work will entail assessment of the effect of ⍺-crystallin knockout on the activation of ER stress and the UPR, as well as the activity of the proteasome arm of misfolded protein degradation. Further studies will also be needed to test potential compensation and synergistic mechanisms using newly developed inducible models to circumvent the developmental issues, such as smaller eye size and early cataract formation, of the whole body double αA/αB knockout that we previously described and thus could not use for the current study [27].

Retinal degenerative diseases, including retinitis pigmentosa, are often associated with an inflammatory state within the retina [57,58,59,60]. In the P23H rat, it was found that microglial infiltration into the subretinal space correlated with the progression of the degeneration, and that activation of systemic inflammation accelerated the loss of photoreceptors [47,61]. Microglial activation in P23H [47] and other rodent models of retinitis pigmentosa caused by rhodopsin mutations [62,63,64] has been described as contributing to the death of the photoreceptors, and interventions that reduce cell death correlate with reduced microglial activation [65] Our data confirm the converse of this postulate, which is that increased cell death as a result of either ⍺A- and ⍺B-crystallin knockout is associated with an increased inflammatory response and increased microglial activation. Future work in our knockout mice will seek to assess whether anti-inflammatory interventions can ameliorate cell death, even in the absence of the ⍺A- and ⍺B-crystallins.

In summary, our data confirm the importance of the ⍺-crystallins in regulating cell death in the P23H mouse model of adRP. Continued work will help define the mechanisms by which this protective effect occurs, and whether these pathways can be exploited therapeutically to improve retinal cell survival and function in these patients.

## 4. Materials and Methods

### 4.1. Animals

All animal procedures were performed in accordance with the Association for Research in Vision and Ophthalmology (ARVO) Statement for the Use of Animals in Ophthalmic and Vision Research and approved by the University Committee on the Use and Care of Animals of the University of Michigan. The Rho^P23H/P23H^ mice were bought from Jackson Lab, and crossed with C57BL/6 mice to produce Rho^P23H/+^ (P23H) mice. Systemic αA-crystallin knock out (AKO) mice and αB-crystallin knock out (BKO) mice were originally provided by Dr. Wawrousek (NIH). Mice were backcrossed over 10 generations and genetically verified using C57bl/6 genetic markers to demonstrate that they were over 96% C57Bl/6. Of note, these animals have normal retinal structure and function. C57Bl/6 were used as wild-type controls. They are referred to as C57 in the text. The Rho^P23H/P23H^ mice were also respectively crossed with AKO mice and BKO mice to produce both α-crystalline deficient and heterozygous P23H mice, namely AKO-Rho^P23H/+^(AKO/P23H) mice and BKO-Rho^P23H/+^ (BKO/P23H) mice. Genotyping through PCR analysis for mice was conducted by Transnetyx, Inc. (Cordova, TN, USA). Mice were bred under a 12-h light/12-h dark cycle at Kellogg Eye Center, University of Michigan.

### 4.2. Spectral Domain Optical Coherence Tomography

Mice were anesthetized with a mixture of ketamine (80 mg/kg, Hopira, Lake Forest, IL, USA) and xylazine (10 mg/kg, NAND, Lake Forest, IL, USA), and pupils were dilated with topical 2.5% phenylephrine (Paragon BioTek, Inc., Portland, OR, USA) and 0.5% tropicamide (AKORN, Lake Forest, IL, USA). Optical coherence tomography (OCT) was performed with a spectral domain optical coherence tomography system (Bioptigen, Inc., Durham, NC, USA) following the application of Systane Lubricant eye drops (9004494-0109, Alcon, Fort Worth, TX, USA) to hydrate cornea. A volume scan on a 1.2 × 1.2 mm square centered on the optic nerve head was performed. Outer nuclear layer (ONL) thickness was analyzed in both superior and inferior of the retina at distances of 250 and 500 µm from the optic nerve head.

### 4.3. Electroretinography

Electroretinography (ERG) responses were detected by a Celeris ERG System (Diagnosys, Lowell, MA, USA) under dim red light to evaluate retinal function. After dark adapted overnight, mice were anesthetized as previously mentioned. Corneal ERGs were recorded from both eyes using gold wire loops mounted in a contact lens electrode (Mayo Corporation, Yamaguchi, Japan) following pupil dilation. The scotopic ERG was recorded at 0.01, 10, 32 log cd s/m^2^ intensity with stimulus of white light. Mice were then light-adapted for 10 min and photopic responses were recorded at 0.01, 10, 32, and 100 cd s/m^2^. Mice body temperature was maintained at 37 °C via a built-in heating board during the experiment. The amplitudes of a-wave and b-wave were measured by Espion V6 software (Diagnosys, Lowell, MA, USA).

### 4.4. Histology and Immunohistochemistry

After euthanasia for mice, a burn (GEMINI Cautery System, GEM5917, distributed by Braintree Scientific, Inc., Braintree, MA, USA) was made at the superior cornea of the eyes to mark their orientation. For paraffin sections, the eyes were enucleated, fixed in 4% paraformaldehyde overnight at 4 °C, and processed by paraffin embedding. Six-µm-thick sections were cut using a Shandon AS325 microtome (Thermo Fisher Scientific, Cheshire, UK). After deparaffinization in xylene and rehydration in ethyl alcohol, sections crossing the optic nerve were stained with hematoxylin and eosin (Fisher Scientific, Hercules, CA, USA) and photographed with a Leica DM6000 microscope. 

For immunofluorescence, two methods to obtain cryosections were optimized to get better staining images. For rhodopsin and m-opsin staining, the eyes were fixed in a manner of freeze substitution as previously mentioned [21]. Firstly, the eyes were shock-frozen in dry ice-cooled liquid propane for 30 s and then kept in methanol containing 3% glacial acetic acid at −80 °C for 48 h, followed by −20 °C overnight. Subsequently, the eyes were shifted to 100% ethanol accompanied with removal of cornea and lens. After 2 h in 100% ethanol, the eyecups were transitioned through ethanol from 90% to 20% at a descending gradient of 10% for 20 min each. The eyes were embedded in mixture of sucrose and embedding medium as mentioned above, and were cut to obtain 10-μm sections. Only sections crossing the optic nerve were used for staining. Frozen sections were rehydrated with PBS and blocked with 5% normal goat serum (NGS) in PBS with 0.1% Triton X-100 (PBST) for 1 h, followed by incubation with primary antibodies (rhodopsin, NBP1-48334; Novus Biologicals, Centennial, CO, USA; m-opsin, AB-5405, Millipore, Burlington, MA, USA) overnight. After washing for three times in PBST, sections were incubated with secondary antibody (Thermo Fisher Scientific, Waltham, MA, USA) for 1 h at RT and mounted with Prolong Gold with DAPI (Invitrogen, P26931, Eugene, OR, USA). Pictures were taken at comparable areas of the sectioned with a fixed detection gain using a confocal microscope (Leica SP5, Leica Corp., Wetzlar, Germany).

For Iba-1 staining, the eyes were fixed in 4% PFA for 2 h at room temperature (RT), followed by removal of cornea and lens. The eyecups were washed by phosphate-buffered saline (PBS) three times and then incubated with 5% (30 min, RT), 10% (30 min, RT) and 20% (overnight, 4 °C) sucrose in PBS. Subsequently, the eyes were embedded in the mixture of 20% sucrose in PBS and Tissue Tek embedding medium (4583, Sakura Finetek, Tokyo, Japan). Thirty-micrometer sections were obtained with a Leica CM3050S cryostat (Leica Corp., Germany). After blocking with 10% NGS in PBS with 0.2% Triton X-100, sections were incubated with Iba1^+^ primary antibody (019-19741, 1:250, Wako Chemicals USA Inc., Richmond, VA, USA) overnight at 4 °C. After washing and incubation with secondary antibody, sections were counterstained with DAPI and mounted with Gel Mount Aqueous Mounting Media (Biomeda, Foster City, CA, USA). Images were taken at comparable regions with fixed gains using a Leica 6000 microscope (Leica Corp., Wetzlar, Germany) or a confocal microscope (SP5; Leica Corp., Wetzlar, Germany).

### 4.5. Western Blot Analysis

For better detection of autophagy flux, the mice received an intraperitoneal injection of leupeptin (40 mg/kg, L2884, Sigma-Aldrich, St. Louis, MO, USA) 4 h before tissue collection [22]. Samples were harvested at the same time of the day, 1 p.m., on account of the fluctuant basic levels of autophagy [66]. After the mice were euthanized, retinal tissue was obtained under a dissecting microscope, as previously described [67]. Extracted proteins were solubilized in RIPA lysis buffer (89900, Thermo Scientific, Darmstadt, Germany) mixed with protease and phosphatase inhibitor, and protein concentrations were quantified using a Dc Protein Assay kit (5000112, Bio-Rad Laboratories, Hercules, CA, USA). Proteins were added to 4–15% Mini-PROTEAN TGX Precast Protein gels (4561086, Bio-Rad Laboratories, Hercules, CA, USA), and then transferred to polyvinylidene fluoride membranes (162-0177, Bio-Rad Laboratories, Hercules, CA, USA). Membranes were blocked with 5% BSA (Sigma-Aldrich, St. Louis, MO, USA) in Tris-buffered saline (TBS; 170-6435, Bio-Rad, Laboratories, Hercules, CA, USA) containing 0.1% Tween 20 for 1 h at room temperature (RT), and incubated with primary antibody overnight at 4 °C. The list of antibodies is as follows: LC3 (4108S, 1:1000, Cell Signaling technology, Danvers, MA, USA), P62/AQATM1 (Novus biologicals, NBP1-48320S;1:1000), Rho 4D2 (NBP1-48334; 1:2000, Novus biologicals, Centennial, CO, USA), GAPDH (AM4300; 1:60,000, Thermo Fisher Scientific, Waltham, MA, USA), RIPK3 (AP7819B, 1:1000, ABGENT, San Diego, CA, USA), pRIPK3 (ab195117, 1:1000, Abcam, Waltham, MA, USA). After three washes for 8 min each, the membranes were then incubated with secondary antibody for 1h at RT. Enhanced chemiluminescence (SuperSignal West Dura Substrate; Thermo Fisher Scientific, Waltham, MA, USA) was used to develop the immunoblots, and signals were detected by cSeries Capture Software (c500; Azure Biosystems, Dublin, CA, USA). Quantitative densitometry was processed by ImageJ software (National Institutes of Health, Bethesda, MD, USA).

### 4.6. Soluble and Insoluble Rhodopsin Fractionation Assay

Soluble and insoluble rhodopsin fractionation assay was performed as previously described [22]. Briefly, retinas were extracted to ice-cold lysis buffer (PBS, pH 7.5, 5 mM EDTA, 1% TX-100) mixed with protease inhibitor (11697498001, Roche, Pleasanton, CA, USA) and kept on ice for 30 min. Samples were centrifugated at 13,000× *g* for 15 min, and the supernatant containing the soluble fraction was collected. For the insoluble fraction, the pelleted pieces were immersed in 1% SDS in PBS for 10 min at RT. After adding additional lysis buffer, the sample was homogenized by a sonicator.

### 4.7. TUNEL Staining

Cryosections crossing the optic nerve from eyes sampled at postnatal 14 days were used for TUNEL staining. DeadEnd Colorimetric TUNEL System (Promega Corporation, Madison, WI, USA) was performed according to the manufacturer’s instructions. TUNEL-positive cells in the outer nuclear layer (ONL) were counted in a masked fashion. Three nonoverlapping sections of each sample were used.

### 4.8. Caspase 8 Activity Assay

Two retinas for each mouse were pooled for detection of caspase 8 activity. After homogenization and centrifugation at 10,000× *g* for 10 min at 4 °C, the protein concentration was quantified by the Dc Protein Assay kit as mentioned above. A luminescent Caspase-Glo^®^ 8 Assay kit (G8201, Promega, Madison, WI, USA) and a plate reader luminometer (Turner Biosystems, Sunnyvale, CA, USA) were used to measure caspase 8 activity.

### 4.9. Real-Time Polymerase Chain Reaction (RT-PCR)

Total RNA from mouse retina was extracted using the RNeasy Mini Kit (74104, Qiagen, Germantown, MD, USA). RNA was quantified using Nanodrop spectrophotometer (Thermo Fisher Scientific, Waltham, MA, USA), and then was converted into cDNA by using the SuperScript III Reverse Transcriptase Kit (18080093, ThermoFisher Scientific, Waltham, MA, USA). The transcript levels of target genes were assessed in triplicate using a CFX384 Real-Time PCR Detection System (Bio-Rad, Laboratories, Hercules, CA, USA). The sequences of specific primers were as follows: caspase 8 (forward 5′-ATGGCGGAACTGTGTGACTCG-3′, reverse 5′-GTCACCGTGGGATAGGATACAGCA-3′); CCL2 (forward 5′-CGTTAACTGCATCTGGCTGA-3′, reverse 5′-AGCACCAGCCAACTCTCACT-3′); IL-1β (forward 5′-GCCCATCCTCTGTGACTCAT-3′, reverse 5′-AGGCCACAGGTATTTTGTCG-3′); IL6(forward 5′-GTGGCTAAGGACCAAGACCA-3′, reverse 5′-TAACGCACTAGGTTTGCCGA-3′); RIPK3 (forward 5′-AGGCTTCTAAAGCGAGTGATGT-3′, reverse 5′-TGAAGTCTTGTCTACCAACTCAGC-3′); RIPK1 (forward 5′-TACCTCCGAGCAGGTCAAAT-3′, reverse 5′-AAACCAGGACTCCTCCACAG-3′); and control Rpl19 (Forward 5′-ATGCCAACTCCCGTCAGCAG-3′, Reverse 5′-TCATCCTTCTCATCCAGGTCACC-3′). Relative levels of target mRNA were normalized with the level of Rpl19 in a comparative cycle threshold method 2^−ΔΔCT^ manner.

### 4.10. Immunohistochemistry and Cell Count on Retinal Whole Mount

The superior cornea was marked by a burn for orientation as mentioned above. Enucleated eyes were fixed with 4% PFA in PBS for 30 min at RT and rinsed twice for 10mins in PBS. After removal of cornea and lens, retina was incised to a four-leaf clover shape, and then carefully separated from the underlying RPE and the rest of the eye cup. Dissected retinas were washed with PBST, blocked with 10% NGS in PBST for 2 h at RT, and incubated with primary antibody (019-9741, 1:250, Wako Chemicals USA Inc., Richmond, VA, USA) at 4 °C for two nights. After repeated washes, retinas were incubated with a mixture of secondary antibody and DAPI at 4 °C overnight. Stained retinas were mounted on slides in an orientation-specific manner for confocal imaging. Images were processed and Z-stacked using software Leica Application Suite X to produce merged images for different layers of the retina [47]. For each sample, images taken from comparable inferior areas of the retina, including at least two regions of the peripheral and two regions of the central retina, were included. The number of Iba1-positive cells in each confocal image at 40x magnification was used for quantification.

### 4.11. Statistical Analyses

Prism 8 software (GraphPad Software, Inc., La Jolla, CA, USA) was used for statistical analysis and generating graphs. Experimental data were expressed as the mean ± standard deviation (SD). Difference between two groups were analyzed by unpaired *t*-test. Comparisons among more than two groups were analyzed by one-way ANOVA followed by Tukey multiple comparison test. Significance was considered at *p* < 0.05.

## Data Availability

The data that support the findings of this study are available from the corresponding author upon reasonable request.
